# Supplementary material for: A Likelihood Approach for Real-Time Calibration of Stochastic Compartmental Epidemic Models
Source: PLoS Comput Biol. 2017 Jan 17;13(1):e1005257. doi: 10.1371/journal.pcbi.1005257 (PMC5240920; doi:10.1371/journal.pcbi.1005257)
Supplement: S1 File — (TAR.GZ) [file pcbi.1005257.s014.tar.gz › HSPH_Online-SI-Revision/PF08/n1-mild/n1-mild_PF_summary.pdf]

peak -8	R0	1/θ_2	inf	inflog	newly	newlylog	Reff	AttackRate
mean	41.	64.9	537.	0.7	496.8	0.6	28.7	73.5
median	38.9	48.4	460.8	0.7	307.5	0.6	26.8	70.
Quantile .1	29.2	24.9	148.8	0.5	123.3	0.4	21.7	51.8
Quantile .9	54.7	122.9	953.3	0.9	972.7	0.9	37.6	101.6
46	42	38						
length	50							

peak -4	R0	1/θ_2	inf	inflog	newly	newlylog	Reff	AttackRate
mean	33.5	67.9	251.2	0.5	173.1	0.4	25.4	73.4
median	32.6	49.7	212.6	0.5	117.6	0.4	25.1	66.1
Quantile .1	23.8	24.	94.7	0.3	53.6	0.3	20.1	44.3
Quantile .9	46.	131.9	499.7	0.7	364.5	0.6	31.1	104.6
45	38	22						
length	50							

peak	R0	1/θ_2	inf	inflog	newly	newlylog	Reff	AttackRate
mean	30.3	67.1	114.9	0.4	78.	0.4	26.5	70.3
median	30.1	51.4	100.1	0.4	56.8	0.4	25.8	61.3
Quantile .1	21.	25.6	60.5	0.3	45.3	0.2	21.	46.1
Quantile .9	40.8	127.2	181.4	0.6	149.9	0.5	30.5	102.4
47	38	23						
length	50							

peak +4	R0	1/θ_2	inf	inflog	newly	newlylog	Reff	AttackRate
mean	30.4	67.6	1014.2	0.5	454.4	0.5	27.6	71.5
median	29.1	52.3	120.7	0.5	71.3	0.4	26.6	64.1
Quantile .1	20.9	25.9	58.8	0.3	49.9	0.3	22.6	45.9
Quantile .9	38.6	136.	336.2	0.7	153.6	0.6	34.2	101.5
45	40	14						
length	50							

peak +8	R0	1/θ_2	inf	inflog	newly	newlylog	Reff	AttackRate
mean	30.7	68.	1797.8	0.7	952.1	0.7	28.5	72.1
median	30.	52.4	143.4	0.6	96.3	0.6	27.4	64.1
Quantile .1	21.3	25.4	66.8	0.4	57.8	0.4	22.3	46.5
Quantile .9	39.5	136.7	1703.6	1.1	808.	0.8	36.2	100.7
47	40	15						
length	50							
